# University Students Eating Habits: Normal Semester vs. Lockdown Period Caused by COVID-19 Pandemic

**DOI:** 10.3390/ijerph191912750

**Published:** 2022-10-05

**Authors:** Mónica Monteiro, Cíntia Ferreira-Pêgo

**Affiliations:** 1 School of Sciences and Health Technologies, Universidade Lusófona de Humanidades e Tecnologias, Av. Campo Grande 376, 1749-024 Lisbon, Portugal; 2 CBIOS—Universidade Lusófona’s Research Center for Biosciences and Health Technologies, Av. Campo Grande 376, 1749-024 Lisbon, Portugal

**Keywords:** COVID-19, lockdown, university students, eating habits, Portugal

## Abstract

In the face of the COVID-19 pandemic, university students’ eating habits may change due to the stress caused by mandatory full lockdown and social isolation, as well as uncertainty about their academic future. An analysis of 332 Portuguese university students from different areas of study was carried out through an online questionnaire to verify if the lockdown period caused by COVID-19 had any influence on the students’ eating habits, as well as if this differed among students from health sciences courses and those from other areas. We found that, compared to a normal semester of classes, during the lockdown period, healthier eating habits were adopted, characterized by a decrease in meal delivery platforms used, self-reported consumption of fast-food, pre-cooked meals, foods rich in sugar and salt, and sugar-sweetened and alcoholic beverages, as well as increased self-reported consumption of vegetables, fruit and legumes, and physical exercise. Thus, although the lockdown led to stress and uncertainty, university students were able to take care of their eating habits and lifestyle, probably as a way of feeling stronger and safer against this disease.

## 1. Introduction

The new coronavirus, recognized as SARS-CoV-2 (Severe Acute Respiratory Syndrome Coronavirus 2) and identified for the first time in late 2019 in the city of Wuhan, China, can cause severe respiratory infections, thus originating the designated pathology COVID-19 (Coronavirus Disease 2019) [1]. On 11 March 2020, after becoming a major threat to global public health, the WHO (World Health Organization) declared COVID-19 a pandemic [2]. To reduce the spread of the virus, a state of emergency was implemented in numerous countries, including Portugal, and, with it, mandatory lockdown and social isolation [3]. Mandatory confinement can be considered an unpleasant experience due to the distance of loved ones, the obligation to stay indoors for a long and indeterminate period, loss of freedom, lack of routine, uncertainty about the state of the disease, and boredom, which can lead to stressful conditions, and, consequently, changes in eating habits [4,5]. Confinement may also lead to an increase in sedentary behaviors, which involve activities with low energy expenditure, performed mainly in a sitting or lying position [6]. Boredom is associated with a greater intake of energy, as well as with the consumption of sweets, since the desire for food rich in sugar stimulates the production of serotonin which, in turn, has a positive effect on mood [3]. Humans are generally sociable beings, especially those of a younger age, such as students, and for this reason, the period of social isolation is also a factor that can influence the eating habits of university students [4]. Additionally, the closure of universities, the teaching of online classes and online evaluations, uncertainty about their academic future, and concern at the economic level of not being able to pay tuition fees due to the loss of the families’ sources of income are factors that influence mental health and are associated with anxiety and stress symptoms in university students during the pandemic [7]. Scientific evidence on the relationship between COVID-19 and nutrition is still scarce, and there is no specific food or dietary supplement that can prevent or assist the treatment of COVID-19 [8]. However, it is known that a healthy diet and an adequate nutritional status have an influence on the immune system and susceptibility to diseases [6]. Therefore, it is extremely important to maintain a healthy and balanced eating routine during this period, reducing a high caloric intake by reducing frequent snacks [6], as well as reducing the consumption of food rich in sugar and salt [8]. In addition, to guarantee the normal functioning of the immune system, the balanced presence of different nutrients in the diet is essential, prioritizing the regular consumption of fruit, vegetables, legumes, and water [8]. Taking all this information into consideration, the main objective of the present study was to observe and compare the eating habits of Portuguese university students during a normal academic semester and the period of lockdown due to the COVID-19 pandemic, as well as to determine whether eating habits differed between students from the health sciences and those from other study areas.

## 2. Materials and Methods

### 2.1. Population and Study Design

The present study consisted of longitudinal analysis. The population studied included Portuguese university students, aged between 18 and 35 years old and from different areas of study. Study areas were divided into two groups: degrees related to the health sciences (such as nursing, medicine, sports sciences, nutrition, pharmacy, etc.), and degrees not related to the health sciences (such as business, law, arts, marketing, enginery, etc.) The final sample consisted of 332 participants. All individuals, before data collection, agreed to participate in the study, giving their informed and written consent. At the beginning of the questionnaire, the objective of the study, the variables to be evaluated, and the maintenance of anonymity of the data were made available to the participants. The present study was performed following the ethical standards laid down in the 1964 Declaration of Helsinki and its later amendments or comparable ethical standards. The Institutional Review Board declared that this study is exempt from ethics committee approval since it was an observational study without any type of intervention and all the data were collected online, and not involving any type of human contact.

### 2.2. Evaluation of Eating Habits

To assess the eating habits of Portuguese university students, self-reported data were collected through a digital questionnaire using the Google^®^ Forms platform. The individuals included in the present analysis were previously recruited for another work (not published) with the main aim of comparing the differences between eating habits during the normal semester and academic evaluations. Taking into consideration the onset of the COVID-19 pandemic, the individuals were subsequently contacted to answer the same questions, now regarding their eating habits during the lockdown caused by the COVID-19 pandemic. The recruitment rate/agreement rate amongst invited universities and students was 95%. Data collection regarding the normal semester occurred between 22 October and 11 December 2019, and for COVID-19 confinement data were carried out between 30 March and 18 May 2020. During this period of lockdown, all lectures were performed online. In addition, walks and outdoor activities were restricted, and only essential activities were allowed, such as going to the grocery store. 

The questionnaire was divided into different sections: the first concerned the general characteristics of the participants (gender, age, body mass, and height, among others); the second focused on students’ eating habits, where the variables evaluated were the use of food delivery platforms; self-reported consumption of fast-food; self-reported consumption of pre-cooked meals; self-reported consumption of snacks and sweets as a stress consolation; the habit of skipping meals; self-reported consumption of vegetables, fruit, legumes, water, sugar-sweetened beverages (SSB), and alcoholic beverages; the habit of eating to relieve stress; the habit of cooking and consuming meals prepared by themselves or by family/housemates; physical exercise routines; and, finally, referring to the comparison of the self-perception of eating habits quality in those two periods. The questions regarding food consumption were based on a validated food frequency questionnaire [9].

### 2.3. Data Analysis

Statistical analysis was performed using the Statistical Package for Social Sciences (IBM SPSS) version 26.0 (SPSS Inc., Chicago, IL). Data were presented as percentages (n) for dichotomous variables and the mean (SD) for continuous variables. The distribution of the selected characteristics between groups was compared using Pearson χ^2^ tests for categorical variables and Student’s t-tests for continuous variables. All statistical tests were two-tailed and the significance level was set at *p* < 0.05. 

## 3. Results

A total of 332 university students (130 from health sciences courses and 202 from other courses) participated in the present study. The general characteristics of the studied population are described in Table 1.

The average age of the participants was 20.20 (SD: 1.81) years old. In terms of body mass and BMI, the averages were 61.30 (SD:10.49) kg and 22.71 (SD: 3.12) kg/m^2^, respectively. In comparison to the study area, there were significant differences in the level of study and the area of residence.

The comparison of food intake and eating habits between the normal semester and the lockdown period due to the COVID-19 pandemic, according to the area of study of the participants, is shown in Table 2.

The self-reported consumption of sugary and salty foods was statistically lower in the period of lockdown in both groups. At the same time, the self-reported consumption of vegetables at least twice per day was significantly higher during the period of lockdown. In contrast, only approximately 44% of students from the two different studies reported consuming at least 3 pieces of fruit per day during the lockdown period. However, it is important to note that there was a slight, but statistically significant increase during the lockdown period in this intake, mainly in those students from areas not related to the health sciences. Significant differences at a statistical level were also observed in the self-reported consumption of legumes at least three times per week, where there was an increase in self-reported consumption during the lockdown period in both study areas. SSB self-reported consumption statistically decreased during the lockdown period in both study areas. Finally, the self-reported intake of alcoholic beverages decreased drastically during the lockdown period for university students in both groups.

Table 3 shows the use of delivery food platforms and the self-reported consumption of fast-food and pre-cooked meals during the normal semester and the lockdown period, and its comparison. 

In general, the use of food delivery platforms was not common, but it was even lower during the period of lockdown, in both groups. The self-reported consumption of fast food was statistically higher during the normal semester compared to the period of lockdown. Finally, the self-reported consumption of pre-cooked meals was significantly lower during the lockdown period, in both groups. 

The influence of emotions and stress on the eating habits and behaviors of university students is detailed in Table 4. 

Regarding care and concern for nutrition a slight, but significant, decrease was found during the period of confinement for both groups. No statistical association was found regarding eating to relieve stress or boredom, or the influence of mood on eating, between the two assessed periods.

Table 5 shows physical exercise undertaken according to area of study and a comparison between the normal semester and the lockdown period. University students reported practicing significantly more physical exercise during the period of lockdown in both groups.

Table 6 shows a comparison of the quality of eating habits between the normal semester and the period of lockdown due to the COVID-19 pandemic, according to the area of study. Statistically significant differences were observed in the period in which students stated to have greater negligence in eating, reporting that was during the period of academic evaluations that the lack of healthy eating habits prevails. Regarding the perception of students’ eating habits between the lockdown and the normal semester, although there are no statistically significant differences, a highest percentage of participants reported having better eating habits during the lockdown caused by the COVID-19 pandemic.

## 4. Discussion

To the best of our knowledge, this work is the first to reveal that Portuguese university students presented healthier self-reported eating habits and healthier lifestyles during the period of lockdown due to the COVID-19 pandemic compared to a normal semester period. These healthier self-reported eating habits were characterized by a decrease in the use of meal delivery platforms, skipping meals, the consumption of fast food, pre-cooked meals, foods rich in sugar and salt, SSB, and alcoholic beverages, as well as increased consumption of vegetables, fruit, and legumes, and physical exercise. In the sample of the study by Yilmaz et al., university students decreased their consumption of takeaway and pre-cooked meals, as happened in our work [10]. The Yilmaz et al. study was concerned with nutrition knowledge and application since the participants related a healthy lifestyle to a greater immune system, which reduced the influence of their mood on eating habits [10]. Another Portuguese study has reported results contradictory to our own, in that the majority of its participants were not careful with their eating habits, increasing the consumption of foods rich in salt, sugar, and/or fat, such as sweets, snacks, fast food, and SSB during the COVID-19 lockdown period [11]. However, 58.2% of the sample from another Portuguese study changed their eating habits for the better, demonstrating an increase in care and concern with nutrition, with water, fruit, and vegetable consumption increasing, while the consumption of takeaway and pre-cooked meals, SSB, and alcoholic drinks decreased [8]. An Italian study also observed a decrease in the consumption of alcoholic beverages during the period of lockdown, which confirms our results [12]. In a further validation of our results, several studies have also observed that a large part of their samples adopted a healthy diet, characterized by increased consumption of fruit and vegetables, legumes, and water during the lockdown period [12,13,14]. Regarding the increase in physical exercise self-reported in our population, an Italian study also found a greater adherence to the practice of physical exercise during lockdown [12], as did a further Portuguese study [11]. Stress and anxiety, due to full lockdown caused by the COVID-19 pandemic, tend to cause changes in eating habits influenced by emotions, thus generating less healthy eating habits [11]. Another major factor that contributes to high levels of stress and anxiety and, consequently, to a less adequate diet, in students is concern and fear for their health and that of their loved ones [15], which may explain these findings. Additionally, the absence of social interaction, especially in young or active individuals could cause stress, anxiety, and even depression. The feelings of isolation might trigger emotional eating to relieve negative feelings [16]. The fact that the participants spent more time at home may have allowed them to have more opportunities to cook homemade meals and think about their diet, especially given the nutritional recommendations that have been made [11]. Another factor refers to the concern with nutrition since individuals are aware that there is a relationship between a healthy lifestyle and an optimal immune system, which may increase the probability of surviving the virus [10]. However, this relation is not clear and no strong scientific evidence proves it. Additionally, the importance of good nutrition is something that matters for general health and longer and better quality of life. Regarding the pandemic situation, the fact that an online questionnaire was used for data collection was quite advantageous, not only making it possible to respect the social distance imposed as a security measure but also making it more widely accepted by university students. However, some limitations should also be assumed. The sample was not heterogeneous, as the group consisting of students from courses in other areas of study was larger than the group of students from the health sciences; gender was also not equally distributed. In addition, the questionnaire used for data collection has not been validated and the data have been self-reported. It is also fair to assume that the previous nutrition and health literacy of students from the health sciences means that they know what to answer, influencing the results of this analysis. Thus, longer follow-up studies should be performed to clarify the relationship between the impact of the pandemic by COVID-19 and the eating habits of Portuguese university students. 

The present work has several implications for public health policy, and several questions must be raised in view of the results. If university students report eating better when in lockdown situations, how might we learn from this to support better nutrition outside of lockdowns? What might the key influencing factors be? Future research lines should take into consideration that university students are a population susceptible to a lot of eating behavior changes that can last for the rest of their adult lives. In this sense, it is important to consider that any social change, such as the pandemic caused by COVID-19 (that is less present nowadays in some areas of the globe) and, consequently, its lockdown, may condition their health and risk of future disease.

## 5. Conclusions

In conclusion, it was during the lockdown caused by the COVID-19 pandemic that Portuguese university students reported adopting healthier eating habits, characterized by a self-reported decrease in the use of meal delivery platforms, meal skipping, the consumption of fast food, pre-cooked meals, foods rich in sugar and salt, SSB, and alcoholic beverages, as well as increased consumption of vegetables, fruit, legumes, and physical exercise. Thus, although the lockdown caused by the COVID-19 pandemic led to an increase in stress and uncertainty, university students were able to take care of their eating habits and lifestyle. 

## Figures and Tables

**Table 1:** General characteristics of the studied population, according to the study area.

	Total Population (n = 332)	Health Sciences (n = 130)	Other Areas (n = 202)	^a^*p*-Value
**Gender, % (n)**				
Men	9.60 (32)	6.90 (9)	11.40 (23)	0.179
Women	90.40 (300)	93.10 (121)	88.60 (179)	
**Age, years**	20.20 (1.81)	20.28 (2.34)	20.15 (1.37)	0.528
**Weight, kg**	61.30 (10.49)	60.65 (9.90)	61.73 (10.85)	0.361
**Height, m**	1.64 (0.08)	1.63 (0.07)	1.65 (0.08)	0.150
**^b^ BMI, kg/m^2^**	22.71 (3.12)	22.70 (3.14)	22.72 (3.11)	0.947
**Smoking habits, % (n)**				
Smoker	14.50 (48)	11.50 (15)	16.30 (33)	0.203
Former smoker	5.70 (19)	3.80 (5)	6.90 (14)	
Never smoked	79.80 (265)	84.60 (110)	76.70 (155)	
**Study level, % (n)**				
Degree	76.20 (253)	63.80 (83)	84.20 (170)	< 0.001
Other levels	23.80 (79)	36.20 (47)	15.80 (32)	
**Area of residence, % (n)**				
Urban	74.70 (248)	80.80 (105)	70.80 (143)	0.041
Rural	25.30 (84)	19.20 (25)	29.20 (59)	

Data expressed as a percentage (n) or the mean (SD) for categorical or continuous variables, respectively. ^a^ *p*-value, for comparisons between groups, was tested by the Student t-test or Pearson’s χ^2^ test, as appropriate. ^b^ Abbreviations: BMI, Body Mass Index.

**Table 2:** Self-reported food intake and eating habits between the normal semester and the lockdown period due to the COVID-19 pandemic, according to the area of study.

	Total Population (n = 332)			Health Sciences (n = 130)			Other Areas (n = 202)		
	Normal Semester	Lockdown Period	*p*-Value ^a^	Normal Semester	Lockdown Period	*p*-Value ^a^	Normal Semester	Lockdown Period	*p*-Value ^a^
**Snacking**	83.4 (277)	83.4 (277)	0.830	83.8 (109)	86.9 (113)	0.713	83.2 (168)	81.2 (164)	0.785
**Skipping meals**	52.7 (175)	37.3 (124)	<0.001	42.3 (55)	33.8 (44)	<0.001	59.4 (82)	39.6 (80)	<0.001
**Intake of pastry and sweets**	79.5 (264)	70.8 (235)	0.002	82.3 (107)	73.1 (95)	0.035	77.7 (157)	69.3 (140)	0.045
**Consumption of fries and savories**	85.2 (283)	63.9 (212)	<0.001	83.1 (108)	66.9 (87)	0.002	86.6 (175)	61.9 (125)	<0.001
**Consumption > 2 vegetables/day**	45.2 (150)	49.7 (165)	<0.001	52.3 (68)	55.4 (72)	0.036	40.6 (82)	46.0 (93)	0.011
**Consumption > 3 fruit/day**	37.0 (123)	44.0 (146)	0.004	41.5 (76)	43.8 (57)	0.287	34.2 (69)	44.1 (89)	0.009
**Consumption > 3 legumes/week**	57.5 (191)	62.3 (207)	0.001	59.2 (77)	64.6 (84)	0.039	56.4 (114)	60.9 (123)	0.017
**Consumption > 1.5 L/day of water**	50.6 (168)	56.6 (188)	0.280	52.3 (68)	63.8 (83)	0.169	49.5 (100)	52.0 (105)	0.815
**Consumption of juices and SSB**	61.1 (203)	46.4 (154)	<0.001	63.1 (82)	46.9 (61)	0.033	59.9 (121)	46.0 (93)	0.012
**Consumption of alcoholic beverages**	63.3 (210)	14.2 (47)	<0.001	60.8 (79)	13.1 (17)	<0.001	64.9 (131)	14.9 (30)	<0.001

Data expressed as percentages (n). ^a^ *p*-value, for comparisons between groups, was tested by Pearson’s χ^2^ test. Abbreviations: SSB, Sugar-Sweetened Beverages.

**Table 3:** Use of delivery platforms and self-reported consumption of external meals between the normal semester and the lockdown period due to the COVID-19 pandemic, according to the study area.

	Total Population (n = 332)			Health Sciences (n = 130)			Other Areas (n = 202)		
	Normal Semester	Lockdown Period	*p*-Value ^a^	Normal Semester	Lockdown Period	*p*-Value ^a^	Normal Semester	Lockdown Period	*p*-Value ^a^
**Use of food delivery platforms**	36.1 (120)	13.0 (43)	<0.001	38.5 (50)	10.0 (13)	<0.001	34.7 (70)	14.9 (30)	<0.001
**Fast-food intake**	85.8 (285)	23.5 (78)	<0.001	86.9 (113)	20.8 (27)	<0.001	85.1 (172)	25.2 (51)	<0.001
**Intake of pre-cooked meals**	61.4 (204)	37.7 (125)	<0.001	62.3 (81)	39.2 (51)	<0.001	60.9 (123)	36.6 (74)	<0.001

Data expressed as percentages (n). ^a^ *p*-value, for comparisons between groups, was tested by Pearson’s χ^2^ test.

**Table 4:** Eating habits influenced by emotions between the normal semester and the lockdown period due to the COVID-19 pandemic, according to the area of study.

	Total Population (n = 332)			Health Sciences (n = 130)			Other Areas (n = 202)		
	Normal Semester	Lockdown Period	*p*-Value ^a^	Normal Semester	Lockdown Period	*p*-Value ^a^	Normal Semester	Lockdown Period	*p*-Value ^a^
**Eat when bored or need to relieve stress**	70.2 (233)	72.6 (241)	0.126	76.9 (100)	76.9 (100)	0.822	65.8 (133)	69.8 (141)	0.116
**Mood influences eating**	81.3 (270)	73.8 (245)	0.146	81.5 (106)	80.0 (104)	0.369	81.2 (164)	69.8 (141)	0.255
**Care and concern for nutrition**	74.7 (248)	73.8 (245)	<0.001	80.8 (105)	80.0 (104)	<0.001	70.8 (143)	69.8 (141)	<0.001

Data expressed as percentages (n). ^a^ *p*-value, for comparisons between groups, was tested by Pearson’s χ^2^ test.

**Table 5:** Physical exercise practice between the normal semester and the lockdown period due to the COVID-19 pandemic, according to the area of study.

	Total Population (n = 332)			Health Sciences (n = 130)			Other Areas (n = 202)		
	Normal Semester	Lockdown Period	*p*-Value ^a^	Normal Semester	Lockdown Period	*p*-Value ^a^	Normal Semester	Lockdown Period	*p*-Value ^a^
**Physical exercise**	34.0 (113)	47.9 (159)	<0.001	33.8 (44)	50.0 (65)	<0.001	34.2 (69)	46.5 (94)	<0.001

Data expressed as percentages (n). ^a^ *p*-value, for comparisons between groups, was tested by Pearson’s χ^2^ test.

**Table 6:** Comparison of the self-perceived quality of eating habits between the normal semester and the period of lockdown due to COVID-19, according to the area of study.

	Total Population (n = 332)	Health Sciences (n = 130)	Other Areas (n = 202)	^a^*p*-Value
**Greater negligence in eating habits**				
Normal semester	8.10 (27)	5.40 (7)	9.90 (20)	0.045
Academic evaluation period	60.20 (200)	67.70 (88)	55.40 (112)	
Lockdown period	18.10 (60)	18.50 (24)	17.80 (36)	
Maintains eating habits equal	13.60 (45)	8.50 (11)	16.80 (34)	
**How do you consider your eating habits during lockdown compared to the normal semester?**				
Better	39.20 (130)	40.00 (52)	38.60 (78)	0.877
Equal	31.60 (105)	30.00 (39)	32.70 (66)	
Worse	29.20 (97)	30.00 (39)	28.70 (58)	

Data expressed as percentages (n). ^a^ *p*-value, for comparisons between groups, was tested by Pearson’s χ^2^ test.

## Data Availability

Not applicable.

## References

[1] WHO (2020). Q&A on Coronaviruses (COVID-19).

[2] Mehta S. (2020). Nutritional status and COVID-19: An opportunity for lasting change?. Clin. Med. J. R. Coll. Physicians Lond..

[3] Muscogiuri G., Barrea L., Savastano S., Colao A. (2020). Nutritional recommendations for COVID-19 quarantine. Eur. J. Clin. Nutr..

[4] Abbas A.M., Kamel M.M. (2020). Dietary habits in adults during quarantine in the context of COVID-19 pandemic. Obes. Med..

[5] Brooks S.K., Webster R.K., Smith L.E., Woodland L., Wessely S., Greenberg N., Rubin G.J. (2020). The psychological impact of quarantine and how to reduce it: Rapid review of the evidence. Lancet.

[6] Naja F., Hamadeh R. (2020). Nutrition amid the COVID-19 pandemic: A multi-level framework for action. Eur. J. Clin. Nutr..

[7] Verma K. (2020). The mental health impact of the COVID-19 epidemic on college students in India. Asian J. Psychiatr..

[8] Direção-Geral da Saúde (2020). REACT-COVID—Inquérito Sobre Alimentação e Atividade Física em Contexto de Contenção Social.

[9] Moreira P., Sampaio D., Vaz De Almeida M.D. (2003). Validade relativa de um questionário de frequência de consumo alimentar através da comparação com um registo alimentar de quatro dias. Acta Med. Port..

[10] Yilmaz H.Ö., Aslan R., Unal C. (2020). The Effect of the COVID-19 Outbreak on Eating Habits and Food Purchasing Behaviors of University Students. Kesmas Natl. Public Health J..

[11] Antunes R., Frontini R., Amaro N., Salvador R., Matos R., Morouço P., Rebelo-Gonçalves R. (2020). Exploring lifestyle habits, physical activity, anxiety and basic psychological needs in a sample of portuguese adults during COVID-19. Int. J. Environ. Res. Public Health.

[12] Di Renzo L., Gualtieri P., Pivari F., Soldati L., Attinà A., Cinelli G., Cinelli G., Leggeri C., Caparello G., Barrea L. (2020). Eating habits and lifestyle changes during COVID-19 lockdown: An Italian survey. J. Transl. Med..

[13] Sidor A., Rzymski P. (2020). Dietary choices and habits during COVID-19 lockdown: Experience from Poland. Nutrients.

[14] Reyes-Olavarría D., Latorre-Román P.Á., Guzmán-Guzmán I.P., Jerez-Mayorga D., Caamaño-Navarrete F., Delgado-Floody P. (2020). Positive and negative changes in food habits, physical activity patterns, and weight status during COVID-19 confinement: Associated factors in the chilean population. Int. J. Environ. Res. Public Health.

[15] Son C., Hegde S., Smith A., Wang X., Sasangohar F. (2020). Effects of COVID-19 on College Students’ Mental Health in the United States: Interview Survey Study. J. Med. Internet Res..

[16] Pop C., Ciomag V. (2021). Impact of COVID-19 lockdown on body mass index in young adults. Phys. Educ. Stud..

